# Predicting childhood overweight and obesity using maternal and early life risk factors: a systematic review

**DOI:** 10.1111/obr.12640

**Published:** 2017-12-19

**Authors:** N. Ziauddeen, P. J. Roderick, N. S. Macklon, N. A. Alwan

**Affiliations:** ^1^ Academic Unit of Primary Care and Population Sciences, Faculty of Medicine University of Southampton Southampton UK; ^2^ Academic Unit of Human Development and Health, Faculty of Medicine University of Southampton Southampton UK

**Keywords:** Childhood obesity, maternal factors, overweight, prediction models

## Abstract

**Background:**

Childhood obesity is a serious public health challenge, and identification of high‐risk populations with early intervention to prevent its development is a priority. We aimed to systematically review prediction models for childhood overweight/obesity and critically assess the methodology of their development, validation and reporting.

**Methods:**

Medline and Embase were searched systematically for studies describing the development and/or validation of a prediction model/score for overweight and obesity between 1 to 13 years of age. Data were extracted using the Cochrane CHARMS checklist for Prognosis Methods.

**Results:**

Ten studies were identified that developed (one), developed and validated (seven) or externally validated an existing (two) prediction model. Six out of eight models were developed using automated variable selection methods. Two studies used multiple imputation to handle missing data. From all studies, 30,475 participants were included. Of 25 predictors, only seven were included in more than one model with maternal body mass index, birthweight and gender the most common.

**Conclusion:**

Several prediction models exist, but most have not been externally validated or compared with existing models to improve predictive performance. Methodological limitations in model development and validation combined with non‐standard reporting restrict the implementation of existing models for the prevention of childhood obesity.

**Abbreviations**
AUROC: area under receiver operating curve
DOHaD: developmental origins of health and disease
IQR: interquartile range
WHO: World Health Organization

## Introduction

The World Health Organization (WHO) has identified childhood overweight and obesity as one of the most serious public health challenges of the 21st century with 42 million children aged under 5 years estimated as overweight globally in 2014 1. Data from the National Child Measurement Programme in England showed that in 2014/2015, 22% of children in Reception (aged 4 to 5 years) and 33% in Year 6 (aged 10 to 11 years) were classified as overweight or obese with children in most deprived areas twice as likely than children in least deprived areas to be obese 2. In 2012, the WHO published a report on population‐based approaches to childhood obesity prevention, which identified improved government structures to support policy and intervention as well as population‐based and community‐based interventions as actions to prevent childhood obesity 3. In 2014, the European Union published a 6‐year action plan on childhood obesity with the goal of contributing to halting the rise in childhood overweight and obesity by 2020. In 2016, the UK Government published a plan for action for tackling childhood obesity with the aim to significantly reduce rates of childhood obesity within the next 10 years by supporting healthier choices in children and engaging communities, schools and industry to make food and drink healthier 4.

There is evidence that the in utero environment induces a response in the foetus, which can lead to enhanced susceptibility for diseases in later life 5. This concept is described as the ‘developmental origins of health and disease (DOHaD)’. Developing foetuses adapt to an adverse in utero environment by undergoing structural, physiological and hormonal changes, which are beneficial for short‐term survival, but at a cost for future health 6, which could be transmitted through generations 7. The ‘maternal resources hypothesis’ suggests that non‐genetic evolution has led to a competitive dominance of adipocytes over other cell types in the acquisition and sequestering of energy in the body, which is maintained by the co‐existence of excess maternal resources and sedentary behaviour during pregnancy leading to continued dysfunction in foetal metabolism 8. Behavioural patterns are transmitted between generations through socially mediated learning 9, and the postnatal environment could affect the behaviour of infants and young children based on that of the primary caregiver 8. Thus, it has been suggested that DOHaD should include all aspects of environment and all sensitive windows (preconception, pregnancy, early childhood and any others yet to be identified) 7.

Hence, the WHO Commission on Ending Childhood Obesity considered it essential to address critical time periods in development including pre‐conception and pregnancy as well as treating children identified as obese 10. The increasing prevalence of obesity in women of reproductive age affects the health of the mother and puts the offspring at risk of developing childhood obesity and its consequences 11. Given the lack of evidence on effective long‐term treatments, the focus of reducing childhood obesity rates should be on prevention 12. Key to an effective prevention strategy is the ability to identify individuals at particular risk. There is increased risk of persistence of childhood weight status into adulthood 13, 14, 15, 16 particularly in children with two obese parents 17, 18, 19 with a meta‐analysis concluding a low probability of weight change without weight loss treatment 20. Although this tracking of childhood body mass index (BMI) to adulthood was weaker in late adulthood 21, the identification of high‐risk populations and intervening as early as possible to prevent the development of overweight and obesity should be a priority 22 because of the increased risk of adult morbidity and mortality associated with overweight and obesity in childhood and adolescence 23. Once high‐risk populations are identified, mathematical models on childhood obesity trajectories that predict energy imbalance including excess energy intake underlying obesity 24, 25 and calculate the magnitude of intervention necessary to achieve change in weight 25 can be used to guide the intervention.

The aim of this study was to systematically review studies of prediction models for childhood overweight and obesity using maternal and/or early life risk factors and critically assess the development and reporting of the methodology used to develop these models.

## Methods

Medline and Embase were searched from their start dates to December 2016 using recommended filters, and the bibliographies and citations of all included studies were hand searched (using Web of Science Core Collection). The outcome considered was overweight and obesity between 1 and 13 years of age. No criteria were defined for overweight and obesity as different criteria can be considered given the age under consideration. The following search strategy was used:





{Pediatric Obesity/ OR Fetal Macrosomia/ OR

[(child or childhood or children or p#ediatric* or infant* or toddler or embry* or prenatal* or neonat*).mp. AND (obes*.mp. OR overnutrition/ or obesity/ or overweight/ OR overweight.mp. OR over weight.mp.)]} AND

[exp causality/ OR ((Reinforc* or Enabl* or predispos*) and factor*).mp. OR (risk* or predict* or causal* or prognos* or causation).mp.] AND

[exp Maternal Behavior/ OR maternal.mp. OR mother*.mp. OR early life.mp.]





### Eligibility criteria

All studies that reported on one or more multivariable prediction models or scores that have been developed for individual estimation of future risk of childhood overweight and obesity were included. Studies that developed, developed and validated or just validated a risk score were not differentiated. The review was limited to studies conducted in humans and published in English. No limits were imposed on study timing or setting.

### Data extraction and critical appraisal

The list of data extraction was based on the CHARMS checklist published by the Cochrane Prognosis Methods Group 26. The Transparent Reporting of a multivariable prediction model for Individual Prognosis or Diagnosis statement was used to assess transparency in reporting 27. N. Z. assessed all articles and extracted the data. Items extracted from studies describing model development included study design, study population and location, number of study participants, outcome and age of outcome if available, method of modelling, method of internal validation (random split of data, bootstrapping or cross‐validation), number of predictors considered and included in the final model, model presentation and predictive performance including measures of discrimination and calibration where available.

For studies describing external model validation alone, items extracted included study design, study population and location, number of study participants and model performance. Predictors were checked to confirm that these were the same as the original model.

We have critically assessed the conduct and reporting of the methods used to develop these risk prediction models. However, a quantitative synthesis of the prediction models' results was not performed as formal methods for meta‐analysis of models are not yet fully developed and was beyond the scope of this review.

## Results

**Figure 1:**
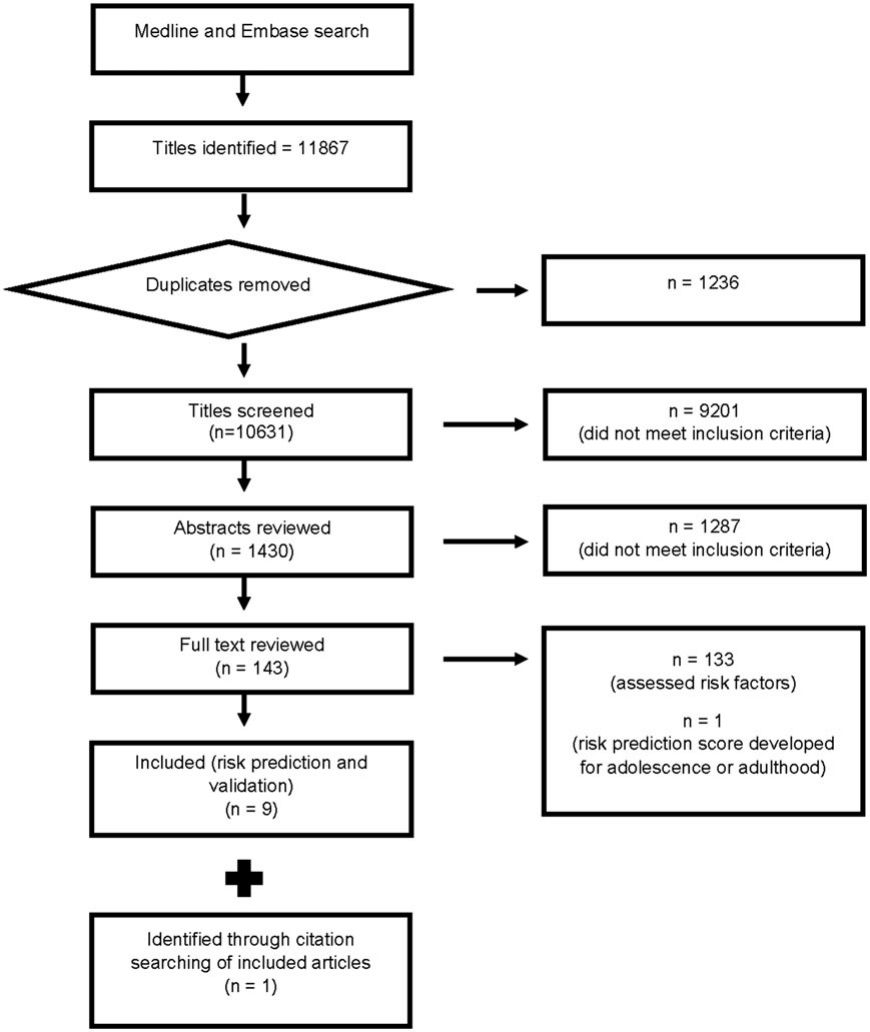
Literature search flow chart.

From the 11,867 articles identified by the search strategy, 143 full articles were reviewed of which nine articles were identified for inclusion in this review (Fig. 1). An additional study was identified through hand searching the citations of the included studies. Eight of the studies developed a risk score, seven of which were internally (six) and/or externally (two) validated in the same publication, and two were external validation studies of two of the eight existing prediction models (Table 1).

**Table 1:** Summary of prediction models in the included studies

Author, year	Druet 2012 34	Manios 2013 31	Manios 2016 37	Morandi 2012 35	Pei 2013 28	Redsell 2016 36	Robson 2016 32	Santorelli 2013 33	Steur 2011 29	Weng 2013 30
*N*, derivation	8,236	2,294	–	4,032	1,515	–	166	1,868	1,687	10,810
*N*, validation	8,236	–	5,946	1,503	757	980		867–880	–	2,703
Country	United Kingdom, Europe, America and Seychelles	Greece	Greece	Finland Validation – Italy, USA	Germany	United Kingdom	USA (Latino cohort)	United Kingdom	The Netherlands	United Kingdom
Design	Meta‐analysis of three birth cohorts	Cross‐sectional with retrospective data collection	Cross‐sectional with retrospective data collection	Prospective birth cohort (Finland, USA); Retrospective cohort (Italy)	Prospective birth cohorts	Prospective birth cohort	Birth cohort	Prospective birth cohorts	Prospective birth cohort	Prospective birth cohort
Outcome	Childhood obesity	Childhood obesity (9–13 years)	Childhood obesity (6–15 years)	Obesity and overweight at 7 years	Overweight at age 10 years	Obesity at age 5 years	Obesity at 5 years	Obesity at age 1 year	Overweight at age 8 years	Overweight at age 3 years
Variables included	4	5	5	6	5	7	10 (full model), 5 (reduced model)	4	6	7
Derivation AUROC	–	0.64	–	0.67 (overweight–obesity), 0.78 (obesity)	–	–	0.84 (full model), 0.82 (reduced model)	0.91	–	0.72
Validation AUROC	0.77	–	0.64	0.70, 0.73	–	0.67 (original model), 0.93 (recalibrated)	–	0.89	–	0.76
TRIPOD	21	19	20	28	23	23	24	29	24	23

AUROC, area under receiver operating curve; TRIPOD, Transparent Reporting of a multivariable prediction model for Individual Prognosis or Diagnosis.

### Study reporting

Using the Transparent Reporting of a multivariable prediction model for Individual Prognosis or Diagnosis 27 reporting recommendation, a median of 23 (interquartile range [IQR], 22 to 24) items out of 37 (31 for derivation or validation alone) were reported suggesting some shortcomings (Table 2). As this review assessed the extent of reporting, authors were not contacted to seek further information.

**Table 2:** TRIPOD items reported in the 10 studies

Title and abstract		TRIPOD item description	Reported
Title	1	Identify the study as developing and/or validating a multivariable prediction model, the target population and the outcome to be predicted	8
Abstract	2	Provide a summary of objectives, study design, setting, participants, sample size, predictors, outcome, statistical analysis, results and conclusions	10
Introduction			
Background and objectives	3a	Explain the medical context (including whether diagnostic or prognostic) and rationale for developing or validating the multivariable prediction model, including references to existing models	9
	3b	Specify the objectives, including whether the study describes the development or validation of the model, or both	10
Source of data	4a	Describe the study design or source of data (e.g. randomized trial, cohort or registry data), separately for the development and validation datasets, if applicable	10
	4b	Specify the key study dates, including start of accrual; end of accrual; and, if applicable, end of follow‐up	10
Participants	5a	Specify key elements of the study setting (e.g. primary care, secondary care and general population) including number and location of centres	10
	5b	Describe eligibility criteria for participants	10
	5c	Give details of treatments received, if relevant	‐
Outcome	6a	Clearly define the outcome that is predicted by the prediction model, including how and when assessed	10
	6b	Report any actions to blind assessment of the outcome to be predicted	0
Predictors	7a	Clearly define all predictors used in developing the multivariable prediction model, including how and when they were measured	8
	7b	Report any actions to blind assessment of predictors for the outcome and other predictors	0
Sample size	8	Explain how the study size was arrived at	10
Missing data	9	Describe how missing data were handled (e.g. complete‐case analysis, single imputation and multiple imputation) with details of any imputation method	4
Statistical analysis methods	10a	Describe how predictors were handled in the analyses	9
	10b	Specify type of model, all model‐building procedures (including any predictor selection) and method for internal validation	8
	10c	For validation, describe how the predictions were calculated	8
	10d	Specify all measures used to assess model performance and, if relevant, to compare multiple models	8
	10e	Describe any model updating (e.g. recalibration) arising from the validation, if carried out	3
Risk groups	11	Provide details on how risk groups were created, if carried out	0
Development vs validation	12	For validation, identify any differences from the development data in setting, eligibility criteria, outcome and predictors	2
Results			
Participants	13a	Describe the flow of participants through the study, including the number of participants with and without the outcome and, if applicable, a summary of the follow‐up time. A diagram may be helpful	6
	13b	Describe the characteristics of the participants (basic demographics, clinical features and available predictors), including the number of participants with missing data for predictors and outcome	7
	13c	For validation, show a comparison with the development data of the distribution of important variables (demographics, predictors and outcome)	1
Model development	14a	Specify the number of participants and outcome events in each analysis	4
	14b	If carried out, report the unadjusted association between each candidate predictor and outcome	1
Model specification	15a	Present the full prediction model to allow predictions for individuals (i.e. all regression coefficients, and model intercept or baseline survival at a given time point)	6
	15b	Explain how to use the prediction model	6
Model performance	16	Report performance measures (with CIs) for the prediction model	7
Model updating	17	If carried out, report the results from any model updating (i.e. model specification, model performance)	1
Discussion			
Limitations	18	Discuss any limitations of the study (such as non‐representative sample, few events per predictor and missing data)	10
Interpretation	19a	For validation, discuss the results with reference to performance in the development data, and any other validation data	3
	19b	Give an overall interpretation of the results, considering objectives, limitations, results from similar studies and other relevant evidence	10
Implications	20	Discuss the potential clinical use of the model and implications for future research	10
Other information			
Supplementary information	21	Provide information about the availability of supplementary resources, such as study protocol, Web calculator and datasets	6
Funding	22	Give the source of funding and the role of the funders for the present study	9

CI, confidence interval; TRIPOD, Transparent Reporting of a multivariable prediction model for Individual Prognosis or Diagnosis.

### Study designs, population and sample size

Most of the studies used data from prospective birth cohorts, and two studies used cross‐sectional studies in childhood with retrospective data collection of maternal and early life factors. All the studies were in high‐income countries with the exception of data from Seychelles in the study that pooled cohort data from three studies.

### Outcomes, number of patients and events

The outcome was overweight (three) 28, 29, 30, obesity (three) 31, 32, 33 or both (two) 34, 35 in the eight included studies that developed a score, and the age at which this was predicted varied from 1 to 13 years of age in children. Sex‐specific and age‐specific BMI was calculated using the International Obesity Task Force 29, 30, 31, 34, 35, Centres for Disease Control 32, WHO 28 and UK90 growth chart 33 criteria and appropriate thresholds for overweight or obesity applied.

The number of participants used to develop the prediction models was clearly reported in all studies. The number of participants was 30,475 from all studies, and the median number was 2,015 (IQR 1,644 to 5,083) across the studies. Six 29, 30, 32, 33, 34, 35 out of eight studies reported the prevalence of the outcome in the study population of which two reported the prevalence of both overweight and obesity (12–23% overweight and 3–32% obesity). Where recorded, the median number of events that was used in model development was 821 (IQR 549 to 1,374) for overweight and 133 (IQR 104 to 170) for obesity.

### Risk predictors

Across the studies analysed, 57 putative predictors (Table 3) with a median of 11 risk predictors (IQR 8 to 19) were considered in the development models. These were defined a priori in six studies 29, 30, 32, 33, 34, 35, identified through previous multivariable regression 31 or defined a priori for maternal predictors and through univariable regression for child predictors 28. Only four of the six studies that defined predictors a priori provided the rationale or references for including these predictors.

**Table 3:** Predictor variables assessed (−) and included (+) in the models

Author, year	Druet 2012 34	Manios 2013 31	Morandi 2012 35	Pei 2013 28	Robson 2016 32, a	Santorelli 2013 33	Steur 2011 29	Weng 2013 30
Gender	+	+	−	+	+	+	+	+
Gestational age	−					−		
Weight change 0–6 months					**+**			
Weight gain 0–1 year (categorized)	−	+						+
Weight gain 0–1 year (continuous)	+					+		
Weight gain 0–5 years (categorized)				−				
Standardized BMI at 60–64 months				+				
Birthweight	+		+	+	**+**	+	+	+
Maternal age					**+**			−
Maternal BMI	+	+	+		**+**	+	+	+
Maternal education		+					−	−
Pre‐pregnancy maternal smoking			−					
Maternal smoking during pregnancy		+	+	+		−	−	+
Maternal occupation			+					
Maternal employment							−	−
Employment in pregnancy								−
Single parenthood/marital status			−					+
Gestational weight gain			−					
Maternal alcohol consumption								−
Maternal feelings of depression								−
Maternal health								−
Maternal diabetes								−
Gestational diabetes						−		
Hospital delivery							+	
Delivery type							−	−
Number of household members			+					
Obesity predisposing single‐nucleotide polymorphisms			−					
Paternal BMI			+				+	+
Paternal education							−	
Paternal employment							−	
Family income (categorized)				+				−
Parental education (categorized)				+				
Solids introduced at < or >6 months					+			−
Exclusive breastfeeding at 4–6 weeks					**+**			
Any breastfeeding at 6 months					+		−	
Ever breastfed in first year								+
Breastfeeding duration								−
Ever formula fed								−
First child/older siblings/number of own children					+		−	−
English language proficiency					+			
Ethnicity						−	−	+
Smoking in the parental house							+	
Living in a highly urbanized environment (≥2,500 address km^−2^)							−	
Maternal vegetable consumption during pregnancy							−	
Premature birth of child							−	
Region of birth							−	
Financial status								−
Child care arrangements								−
Unhappy when feeding interrupted								−
Makes a fuss going to sleep								−
Makes a fuss after waking								−
Upset when not getting things								−
Does the infant sit up?								−
Does the infant stand?								−
Does the infant grab objects?								−
Does the infant hold objects?								−
Can the infant walk?								−

a is **+** included in both full and reduced model and + included in full model only.

BMI, body mass index.

Twenty‐five predictors were included in the final risk prediction models. However, 18 of these predictors were only included in one risk score model. The final reported prediction models included a median of six (IQR 5 to 6) predictors with maternal pre‐pregnancy BMI, birthweight and infant gender included in seven out of eight scores (Table 3). Two studies assessed risk at birth (using preconception, antenatal and birth factors) 29, 35 whereas other scores incorporated weight gain in the first year of life 30, 31, 32, 33, 34 predicting risk from the age of 12 months and over or childhood age‐adjusted and sex‐adjusted BMI at 5 years of age 28 to predict risk at 10 years of age.

### Treatment of continuous risk predictors

Four (50%) risk prediction models retained continuous predictors as continuous 28, 29, 32, 35, two (25%) categorized or dichotomized all continuous predictors and one (12.5%) retained some continuous predictors as continuous and categorized some predictors 33. It was unclear how continuous risk predictors were treated in one study but a categorical score chart developed, so it is likely that all continuous variables were categorized or dichotomized 30.

### Missing data

Four studies only included cases with complete data in model development 28, 29, 33, 34, two studies carried out multiple imputation 32, 35 and one study did not report the presence or handling of missing data 31. The remaining study included participants with full anthropometric data at follow‐up when outcome was assessed, but it is unclear if there were missing data at previous data collection points and how this was handled 30.

One of the studies that carried out multiple imputation had on average 1.7% (range 0 to 11.4%) 35 missing data for each predictor whereas 17% of the other study 32 participants had missing data for at least one predictor. Two of the studies that carried out complete case analysis; 23.8% 29 and 27.2% 28 of the sample were excluded because of the missing data, but it is unclear what percentage of sample was excluded for missing data alone in the other studies 33, 34.

### Model building

Six (75%) studies used automated variable selection (stepwise, backward deletion) to derive the final predictive model 29, 30, 32, 33, 34, 35.

All studies were clear on the method used to develop the prediction model – logistic regression was used in seven studies 29, 30, 31, 32, 33, 34, 35 whereas linear regression was used in one study 28. One study had selected predictor variables based on previous multivariable logistic regression analysis and only carried out univariable logistic regression to assign integer values to the categories of risk predictor variables without any further modelling 31. Two models 29, 33 included interaction terms whilst modelling whereas there was no mention of interaction terms whilst modelling in the other studies.

### Predictive performance

Model performance was assessed in all studies, seven of which used area under the receiving operator curve (AUROC) in either the derivation, validation or both cohorts. The other study tested for specificity and predictive value alone 28. Although model performance was assessed and validated in all studies, only one study reported change in regression co‐efficient post validation and updating the model 29. Two studies from the UK used data from the same birth cohort (Avon Longitudinal Study of Parents and Children) for validation of the same outcome but at different ages (two 33 and five 36 years). Model development AUROC ranged from 0.64 to 0.91 (median 0.78, IQR 0.70 to 0.81). The AUROC of 0.91 was replicated in internal validation using bootstrapping and only decreased to 0.89 on external validation 33.

Three studies 29, 32, 35 carried out Hosmer–Lemeshow tests to test calibration, two of which did so during model development both achieving *p* > 0.5. All studies assessed model classification (sensitivity and specificity) although one study 31 did not present positive and negative predictive values.

### Internal validation

With the exception of two, all studies internally validated the models by random split of data 30, 34, random split followed by cross‐validation 28 or bootstrapping 29, 32, 33. Of the studies that did not internally validate the model, one validated the model externally in two separate cohorts 35 whereas the other was externally validated in a subsequent publication with overlapping authors in the development and validation papers 31, 37. Additionally, one of the studies that internally validated the model using random split was also externally validated in a subsequent publication by the same authors 30, 36. Model validation AUROC ranging from 0.75 to 0.91 (median 0.78, IQR 0.77 to 0.81) was achieved, and the original model was updated in one study only 29. Of the studies that carried out Hosmer–Lemeshow test for calibration, one did not report the exact *p* value, but that *p* > 0.5 was achieved 32 whereas the other achieved *p* = 0.30 on recalibration post validation 29.

### External validation

Only four of eight models have been externally validated – once for three models all of which used data from the same country for validation 33, 36, 37 and twice for one model that was developed in Finland and validated in Italy and USA 35. Of the models validated using data from the same country, two studies calculated AUROC, which were 0.89 36 and 0.67 36. The only study that externally validated the model in two countries other than that in which it was developed 35 found that AUROC (0.70, confidence intervals 0.63 to 0.77) and calibration (Hosmer–Lemeshow *p* = 0.12) were satisfactory in one population, but although AUROC (0.73, confidence intervals 0.67 to 0.80) was satisfactory in the other, calibration (Hosmer–Lemeshow *p* = 0.02) was not. The predictors and model were then tailored to these populations by carrying out a replication analysis using stepwise logistic regression such that calibration achieved satisfactory levels. The initial model developed in Finland included six risk factors and reduced to three and five for the Italian and US cohort, respectively, with only two factors remaining consistent across all three models (maternal and paternal BMI). Ethnicity was introduced in the risk prediction score for the USA, and this was primarily because the birth cohort in Finland had high ethnic homogeneity. One of the external validation studies 36 also developed a recalibrated model using multivariable logistic regression to apply a recalibrated algorithm reflecting the characteristics of the validation cohort, imputed model for missing risk factor prediction and a recalibrated imputed model, which incorporated the two. This led to an increase in discrimination compared with the original model from 2% in the recalibrated to 25% in the recalibrated imputed model.

### Model presentation

The complete regression formula (including all regression coefficients) was presented in six studies 29, 30, 32, 33, 34, 35, and two of these studies provided a decision rule/score chart or risk score algorithm 29, 30. Of the remaining two studies, one provided the regression coefficients 28 whereas the other only provided a score chart 31.

## Discussion

To our knowledge, this is the first systematic review to examine prediction models for childhood overweight and obesity. Eight studies that developed prediction models were identified; however, four of these prediction scores have been externally validated once or twice, and there is no evidence of further validation or validation in populations outside of those in which this was developed. Additionally, new models have been developed with no evidence of comparison with already existing models, and none of the models have been compared with each other to assess predictive performance. There were inadequacies identified in reporting of the methodology of development of risk prediction models, and there is no evidence of implementation of the risk scores. Whilst there is clear overlap between risk factors included in the prediction models, no single risk factor has been included in all prediction models with maternal pre‐pregnancy BMI, infant gender and birthweight being the most commonly included. Thus, it is difficult to recommend the use of any one score, as there are no consistent predictors, no comparison between models and the outcome has been variable and predicted at different ages through childhood up to 13 years of age.

The question of predictors considered for inclusion in the model also needs to be considered. Although not included in the final prediction model, several predictors around infant temperament were considered. These are self‐reported by parents and highly likely to be subjective. Additionally, these factors were identified a priori based on a previous systematic review, but the conclusion of the review was that the evidence was inconclusive because of limited number of studies 38.

Thirteen of the 25 risk factors identified were preconception, and thus, some of these could prove impactful in planned pregnancies such as maternal and paternal BMI whereas others are non‐modifiable such as ethnicity. Although factors such as maternal education, occupation and income are modifiable, it is difficult to do so. Maternal smoking during pregnancy and hospital delivery were the only two antenatal risk factors identified and included in risk prediction. Eight of the 10 early life risk factors identified can be broadly classified into weight gain particularly in the first year of life and breastfeeding including weaning both of which are modifiable. The other two risk factors were gender and birthweight, of which gender is non‐modifiable but birthweight can be monitored and is considered modifiable by factors known to affect foetal growth 39.

Some key aspects of multivariable model development and validation need to be considered. These include handling missing data, method of treatment of continuous variables, selecting variables for inclusion in the model and methods of validation including assessing discrimination and calibration 40. Missing data were identified in most studies, which can introduce bias if inappropriately handled, thus impeding the construction of a valid prediction model 41. Multiple imputation minimizes the effect of missing data provided that data are missing at random 42 and enables the use of all available data but was only performed in 25% of studies included in this review. All other studies excluded participants with missing data, which is an acceptable approach only if the amount of missing data are small 43; however, these studies did not provide any indication of how much data were missing per individual and per variable to enable readers to reach their own judgement of the validity of the prediction.

At least three prediction models categorized some or all continuous variables for inclusion in the model. However, discarding information through categorization of continuous variables to estimate a continuous relationship between a predictor variable and risk has been shown to lead to a substantial loss of power and precision 44, thus reducing the efficiency of the analysis with increased probability of biased estimates 45 and Type 1 46. In addition, a model that categorizes continuous variables is unrealistic as individuals close to but on opposite side of the category cut‐point will be characterized as having very different outcome when a very similar outcome is more likely 47. It is recommended that continuous predictors are retained as continuous and suitable functions such as fractional polynomial are used 47, 48. Although this is true from a methodological point of view, the clinical practice in terms of implementation of any score needs to be considered. For example, the National Institute for Health and Care Excellence in the UK recommends action before, during and after pregnancy in women with BMI greater than 30 49. Thus, including this categorization could make the prediction rule easier to incorporate into clinical practice.

Although predictors shown to have little effect on the outcome should not be included in the prediction, the method of selection of predictor variables for inclusion is crucial. The majority of studies (75%) used an automated variable selection method, which increases the likelihood that variables that do not truly predict the outcome will be identified as a predictor 50. This is because it is a data‐driven approach that cannot account for clinical relevance leading to biased regression estimates and poor predictions as true predictors could be excluded because of lack of power 51, 52. It also leads to loss of information due to inclusion of variables based on a binary decision. It has been suggested that a more reasonable reduction of variables using automated selection procedures could be achieved by using a liberal selection criteria such as *p* = 0.50 52 instead of 0.05, which is more commonly used and has been used in all the prediction models included in this review that used this procedure. It could also be important to retain predictors known to be important from literature but does not achieve statistical significance in the model development dataset 51.

Once developed, the performance of a model needs to be evaluated to demonstrate usability. Although a biased model could provide useful clinical separation into groups if the predictor information entered into the model is strong 53, evidence is needed that the model performs well in populations other than that in which it was developed 54. Validation can be internal or external using a completely different sample, thus also examining the generalizability of the model 54. Six studies (75%) internally validated the model through random split of the dataset (two), random split and cross‐validation (one) or bootstrapping (three). Four studies (50%) externally validated the model, only one of which externally validated the model in cohorts from different countries. This was followed by replication analysis to rebuild the model in these two cohorts resulting in only two predictors being retained across all three models in this study (maternal and paternal BMI). As the use of random split sample decreases the precision of estimates and increases the frequency of missing important independent variable 55, there is limited value in doing so unless the sample size is particularly large 51. A non‐random or chronological split has been suggested as a more precise approach, but internal methods such as bootstrapping and cross‐validation remain more informative 53.

This review has been carried out with a systematic approach, thus identifying all studies that have developed and/or validated a risk prediction model for childhood overweight and obesity. However, heterogeneity exists at many levels particularly the outcome (overweight, obesity or both) under consideration and age at which outcome is predicted. This heterogeneity combined with the deficiency of external validation limits the applicability of these scores. Additionally, poor reporting in aspects of development of the prediction models was observed with insufficient detail on steps involved in model building. Risk prediction models have nearly all been developed or validated in developed countries, but almost half and one‐quarter of the estimated 42 million overweight children under the age of 5 years live in Asia and Africa, respectively 1. Models tailored to these countries are important, as associations are known to vary between ethnic groups.

## Conclusion

Despite the existence of several models for the prediction of childhood overweight and obesity, most have not been externally validated or compared with existing models to assess predictive performance. Moreover as the outcome has been predicted at different ages, it may not be possible to combine or compare all models against each other. This review also highlights methodological limitations in model development and validation combined with non‐standard reporting, thus limiting the usability of these prediction models.

There remains a need to develop new methods for combining findings from existing prediction models and develop prediction models using robust methods of development followed by external validation and recalibrating to populations, which would then enable assessment of impact of the implementation of the score.

## Funding

This work is supported by a University of Southampton Primary Care and Population Sciences PhD studentship (to N. Z.), the Academy of Medical Sciences and the Wellcome Trust (Grant no. AMS_HOP001\1060 to N. A. A.). N. A. A. is also in receipt of research support from and the National Institute for Health Research through the NIHR Southampton Biomedical Research Centre.

## Conflict of interest statement

The authors have no conflicts of interest to declare.
